# C-reactive protein-albumin-lymphocyte index and survival outcomes in renal cell carcinoma: a systematic review and meta-analysis

**DOI:** 10.3389/fonc.2026.1994071

**Published:** 2026-10-27

**Authors:** Yu Wei, Lingyan Mai, Xiuli Zhang, Yingying Zhang, Yuxuan Lin

**Affiliations:** 1 Department of Pharmacy, Jiangbin Hospital of Guangxi Zhuang Autonomous Region, Nanning, China; 2 Department of Pharmacy, Guangxi Hospital Division of The First Affiliated Hospital, Sun Yat-sen University, Nanning, China

**Keywords:** biomarker, CALLY index, meta−analysis, prognosis, renal cell carcinoma

## Abstract

**Background:**

The C-reactive protein-albumin-lymphocyte (CALLY) index is a composite biomarker integrating systemic inflammation, nutritional status, and immune competence. Although its prognostic value has been investigated in several malignancies, its clinical relevance in renal cell carcinoma (RCC) remains incompletely defined. We conducted a systematic review and meta-analysis to evaluate the association between the CALLY index and survival outcomes in patients with RCC.

**Methods:**

PubMed and Embase were systematically searched from database inception to August 2026 for cohort studies evaluating the prognostic value of the CALLY index in RCC. Hazard ratios (HRs) and 95% confidence intervals (CIs) for progression-free survival (PFS) and overall survival (OS) were extracted and pooled using random-effects models. Subgroup analyses were performed according to the clinical populations represented by localized/clear cell RCC (ccRCC) and metastatic/mixed-histology RCC.

**Results:**

Five retrospective cohort studies comprising 587 patients were included. Across clinically heterogeneous RCC populations, a low CALLY index was associated with worse PFS (pooled HR = 2.89, 95% CI: 1.82–4.58; *I²* = 24.7%) and OS (pooled HR = 2.32, 95% CI: 1.61–3.35; *I²* = 0%). The 95% prediction interval included the null value for PFS (0.84–9.98) but not for OS (1.30–4.17). Subgroup analyses comparing localized/ccRCC with metastatic/mixed-histology populations did not identify statistically significant differences between subgroups for PFS or OS.

**Conclusion:**

A low CALLY index was associated with poorer PFS and OS across the heterogeneous RCC populations represented in the available retrospective studies. These findings support the potential role of CALLY as an adjunctive prognostic biomarker but do not establish stage-specific or treatment-specific effects. Prospective multicenter studies using standardized measurement protocols and externally validated cut-offs are needed to determine its incremental prognostic value and clinical utility.

**Systematic review registration:**

https://www.crd.york.ac.uk/PROSPERO/view/CRD420261484115, identifier CRD420261484115.

## Introduction

Renal cell carcinoma (RCC) accounts for approximately 2% of all cancers worldwide and remains one of the most lethal urological malignancies (1, 2). Its incidence has increased globally, partly reflecting the widespread use of abdominal imaging and the consequent detection of incidentally identified renal masses (1). Surgical resection remains the cornerstone of treatment for localized disease; however, a substantial proportion of patients experience recurrence after nephrectomy, particularly those with locally advanced tumors (1). Patients presenting with metastatic RCC have substantially poorer long-term outcomes despite major advances in systemic therapy (1). Moreover, considerable variation in clinical outcomes persists even among patients with similar pathological characteristics, underscoring the biological heterogeneity of RCC and the limitations of conventional clinicopathological parameters alone, including TNM stage, histological grade, and tumor size, in accurately estimating individual prognosis (3, 4). These considerations have prompted increasing interest in accessible and reproducible biomarkers that may complement established risk stratification approaches.

Systemic inflammation, nutritional status, and host immune competence are increasingly recognized as important determinants of cancer progression and treatment response (5, 6). Chronic inflammation can promote tumor-cell proliferation, angiogenesis, invasion, and immune dysregulation, whereas malnutrition and lymphopenia may reflect impaired host antitumor capacity. These processes may be particularly relevant in RCC, a malignancy characterized by complex interactions among angiogenic signaling, tumor metabolism, inflammation, and immune surveillance (7). Several blood-based biomarkers, including the neutrophil-to-lymphocyte ratio (NLR), platelet-to-lymphocyte ratio (PLR), systemic inflammation index (SII), and prognostic nutritional index (PNI), have been associated with survival outcomes in RCC (8). However, most individual biomarkers capture only one component of the host response and may therefore provide an incomplete representation of the interplay between systemic inflammation, nutritional reserve, and immune competence.

The C-reactive protein-albumin-lymphocyte (CALLY) index is a composite biomarker integrating three routinely available laboratory parameters: C-reactive protein (CRP), serum albumin, and absolute lymphocyte count (9). The CALLY index is calculated as [serum albumin (g/dL) × absolute lymphocyte count (cells/μL)]/[CRP (mg/dL) × 10^4^], thereby integrating systemic inflammation, nutritional status, and immune competence into a single measure (9). Although this scaled formulation has been used in several RCC studies, variations in the numerical scaling convention have also been reported, which complicates direct comparisons of cut-off values across studies (10–14). The index was initially introduced as a prognostic biomarker in hepatocellular carcinoma and has subsequently been investigated in several other malignancies (9, 15). Recent studies have also examined its prognostic relevance in RCC, reporting associations between lower CALLY values and poorer survival in both localized and metastatic disease (10–14). Nevertheless, the available RCC evidence remains limited by small sample sizes, heterogeneous patient populations, differences in treatment settings and timing of blood sampling, and variation in CALLY cut-off determination. Importantly, the timing of CALLY assessment varies across RCC studies, ranging from the early postoperative period after nephrectomy to pretreatment assessment before systemic therapy in metastatic disease. These clinical and methodological differences may contribute to variation in the reported prognostic associations and complicate interpretation of the existing evidence.

To date, the prognostic evidence for the CALLY index in RCC has not been systematically synthesized with explicit consideration of these differences. Therefore, we conducted a systematic review and meta-analysis of cohort studies evaluating the association between the CALLY index and survival outcomes in patients with RCC. We further explored the consistency of the association across clinically distinct study populations and considered the potential implications of differences in disease stage, histological composition, treatment setting, timing of CALLY assessment, and formula scaling.

## Methods

### Study registration

This systematic review and meta-analysis was conducted in accordance with the Preferred Reporting Items for Systematic Reviews and Meta-Analyses (PRISMA) 2020 statement (16). The study protocol was prospectively registered in the International Prospective Register of Systematic Reviews (PROSPERO; identifier: CRD420261484115).

### Data sources and search strategy

PubMed and Embase were systematically searched from database inception to August 20, 2026 for studies evaluating the prognostic value of the CALLY index in patients with RCC. The search strategy combined controlled vocabulary terms and free-text terms covering two principal domains: (1) the exposure of interest and (2) the disease of interest. No language or publication-date restrictions were applied. The final searches were conducted in PubMed and Embase on October 10, 2026. The exact search dates and complete database-specific search strategies are provided in **Supplementary Table 1**.

PubMed and Embase were selected to provide broad and complementary coverage of biomedical and clinical literature relevant to RCC and prognostic biomarkers. In addition, the reference lists of included studies and relevant reviews were manually screened to identify potentially eligible studies not retrieved by the electronic searches.

### Eligibility criteria

Studies were eligible if they met the following PICOS criteria:

Population (P): Adults (≥18 years) with histopathologically confirmed RCC of any stage.
Exposure (I): CALLY index measured from peripheral blood samples, regardless of the reported cut-off value or timing of measurement, including preoperative, postoperative, or pretreatment assessments.
Comparator (C): Lower *versus* higher CALLY index groups according to the cut-off defined in each original study. For consistency of interpretation, effect estimates were oriented as low *versus* high CALLY index when necessary.
Outcomes (O): Progression-free survival (PFS) and/or overall survival (OS) reported as HRs with 95% confidence intervals (CIs).
Study design (S): Prospective or retrospective cohort studies.

Studies were excluded if they met any of the following criteria: (1) insufficient information to extract HRs and 95% CIs for survival outcomes; (2) duplicate publications or overlapping study populations, in which case the study with the largest sample size or most complete dataset was retained; (3) case reports, case series, reviews, editorials, conference abstracts without sufficient full-text data, or animal and *in vitro* studies; or (4) studies evaluating the CALLY index only in non-RCC malignancies or mixed cancer populations without RCC-specific data.

### Study selection and data extraction

Two reviewers independently screened titles/abstracts and full-text articles and independently extracted the data. Agreement for study selection was assessed using percentage agreement and Cohen’s kappa, whereas agreement for data extraction was assessed by comparing the independently extracted data. Disagreements were resolved through discussion or, when necessary, consultation with a third reviewer.

For each eligible study, data were independently extracted by two reviewers using a standardized data collection form. Extracted information included study characteristics, patient characteristics, CALLY-related characteristics, and outcome data. Study characteristics included first author, publication year, study design, and sample size. Patient characteristics included histological subtype, disease stage, and treatment received. CALLY-related characteristics included the reported formula, numerical scaling convention, cut-off value, cut-off determination method, and timing of blood sampling. Outcome data included HRs and 95% CIs for PFS and OS from univariable and multivariable Cox regression analyses, together with the covariates included in multivariable models.

When multiple HR estimates were reported for the same outcome, the most fully adjusted multivariable estimate was preferentially extracted to minimize potential confounding. If only a univariable estimate was available, the univariable estimate was retained and this limitation was documented.

### Quality assessment

The methodological quality of each included cohort study was independently assessed by two reviewers using the Newcastle–Ottawa Scale (NOS) (17). The NOS evaluates three domains: selection of study participants (0–4 stars), comparability of study groups (0–2 stars), and ascertainment of outcomes (0–3 stars). Studies with a total score of ≥7 stars were considered high quality, those scoring 4–6 stars were considered moderate quality, and those scoring ≤3 stars were considered low quality. Discrepancies were resolved through consensus or consultation with a third reviewer.

### Statistical analysis

All statistical analyses were performed using R version 4.5.1 with the *meta* package. Associations between the CALLY index and survival outcomes were quantified by pooling HRs and 95% CIs from individual studies. To ensure consistent interpretation across studies, all HRs were oriented to a common reference direction of low versus high CALLY index, such that an HR >1 indicated a higher risk of disease progression or death among patients with a low CALLY index. For studies reporting HRs in the opposite direction, the HR was converted by taking its reciprocal, and the corresponding 95% CI was recalculated by taking the reciprocal of the original upper and lower limits.

Given the anticipated clinical and methodological heterogeneity among studies, including differences in disease stage, treatment setting, timing of CALLY assessment, cut-off values, and numerical scaling conventions, pooled estimates were calculated using a random-effects model. Between-study variance was estimated using the Sidik–Jonkman (SJ) estimator. The between-study variance (τ²) was reported alongside *I²*. In addition, 95% prediction intervals were calculated using a t-distribution-based method with the Sidik–Jonkman estimator for between-study variance, to describe the range of effects that may plausibly be expected in a future comparable study.

Pre-specified subgroup analyses were performed according to the clinical populations represented by the included studies. Studies were categorized as localized/ccRCC versus metastatic/mixed-histology RCC. Because disease stage and histological composition were completely concordant across the available studies—localized cohorts consisted exclusively of ccRCC, whereas metastatic cohorts included mixed histologies—these characteristics could not be statistically disentangled. Therefore, subgroup analyses were interpreted as comparisons between localized/ccRCC and metastatic/mixed-histology populations rather than as independent effects of disease stage or histology. Subgroup differences were assessed using a *Q*-test-based approach.

Sensitivity analyses were conducted using leave-one-out analysis, sequentially omitting one study at a time to evaluate the influence of individual studies on the pooled estimates. Publication bias and small-study effects were assessed visually using funnel plots. Egger’s regression test was additionally performed as an exploratory analysis. Because fewer than 10 studies were available for each outcome, the results of funnel plots and Egger’s test were interpreted cautiously and were not considered definitive evidence for or against publication bias.

All statistical tests were two-sided, and a *P* value <0.05 was considered statistically significant. For Cochran’s *Q* test, *P* < 0.10 was considered indicative of statistically significant heterogeneity because of its limited statistical power.

## Results

### Study selection

A total of 32 records were identified through systematic searches of PubMed and Embase. After removal of five duplicates, 27 records underwent title and abstract screening, of which 21 were excluded because they were irrelevant to the research question or clearly did not meet the eligibility criteria. The remaining six articles underwent full-text assessment. One study was subsequently excluded because it reported disease-free survival (DFS) as its only survival endpoint and did not report HRs for our prespecified outcomes of PFS or OS (18). Ultimately, five studies were included in the meta-analysis (10–14). The study selection process is summarized in the PRISMA flow diagram (**Figure 1**). Agreement between the two reviewers was 100% for study selection (Cohen’s κ = 1.0) and data extraction. No discrepancies required third-party adjudication.

**Figure 1:**
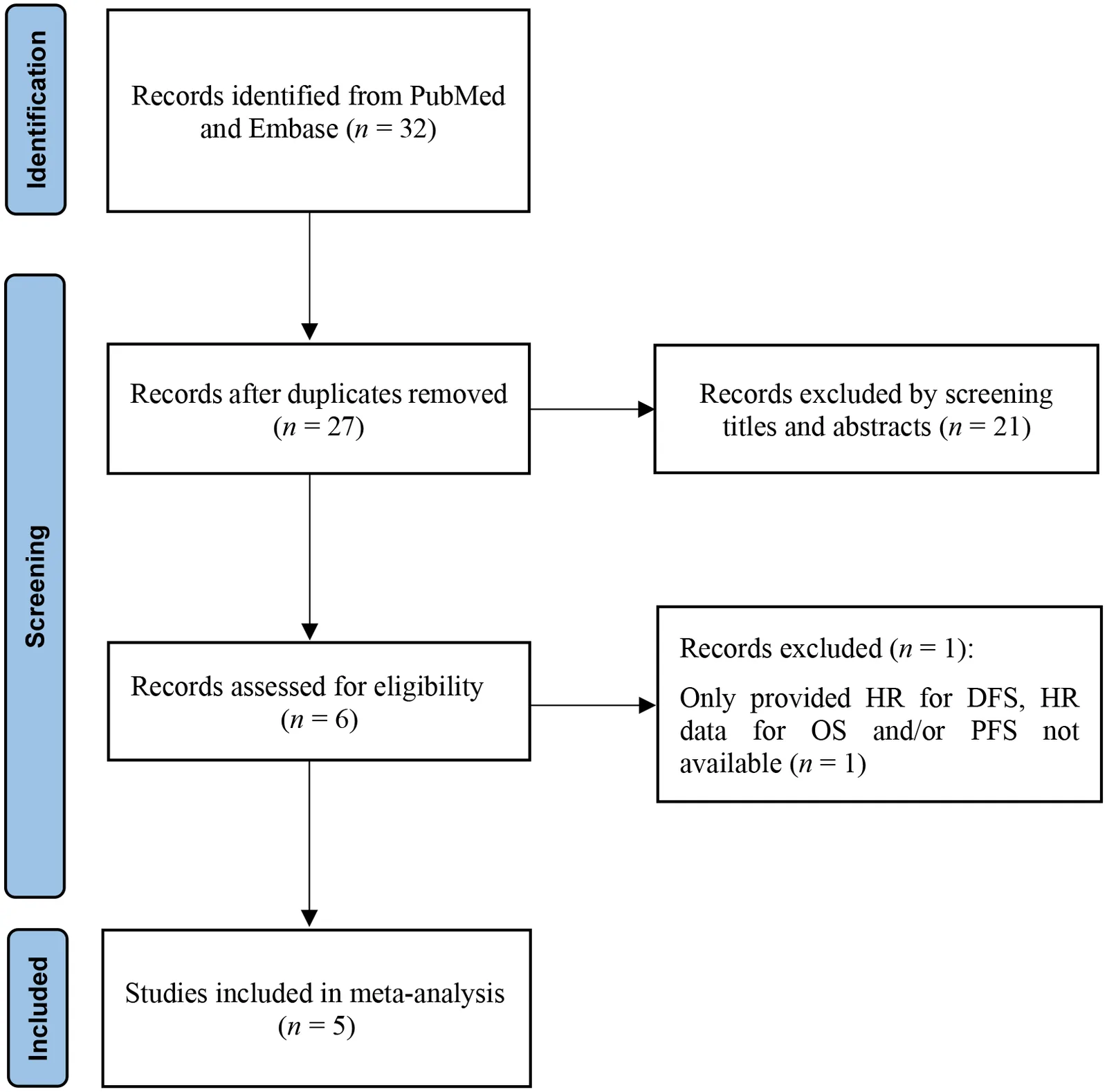
PRISMA flow diagram of study selection.

### Characteristics of the included studies

Five retrospective cohort studies published between 2024 and 2026 were included, comprising a total of 587 patients, with sample sizes ranging from 68 to 253. Two studies were conducted in Japan and three in Turkey. Two studies evaluated localized RCC, whereas three focused exclusively on metastatic RCC. The localized cohorts consisted entirely of ccRCC, whereas the metastatic cohorts included mixed histological subtypes. Treatment settings differed according to disease stage. Patients with localized disease underwent partial or radical nephrectomy, whereas patients with metastatic disease received systemic therapies, including VEGF-targeted tyrosine kinase inhibitors and immune checkpoint inhibitors.

Both studies by Hirata et al. (10, 12) were conducted at Yamaguchi University Hospital, Japan, during the same recruitment period (October 2005 to September 2023). The 2024 study reported a parent cohort of 378 patients with ccRCC, but the effect estimates extracted for our meta-analysis were based exclusively on the 80 patients with pT3 disease. The 2025 study included 253 patients with pT1/T2N0M0 ccRCC. Thus, although the two reports originated from the same institution and recruitment period, the patient subsets contributing to the respective meta-analytic estimates were mutually exclusive by pathological stage. No patient contributed to both effect estimates.

CALLY was derived from peripheral blood parameters in all studies, but the reported numerical scales and cut-off values differed. The two studies by Hirata et al. (10, 12) used a formulation without the (10^4^) scaling factor, whereas the other studies used a formulation incorporating (10^4^) in the denominator (11, 13, 14). When the underlying laboratory units and calculation procedures are equivalent, these formulations differ only by a fixed numerical scaling factor. This scaling changes the numerical values and corresponding cut-off values but does not alter the relative ranking of patients. Therefore, when patient classification remains unchanged, the different numerical scales primarily affect the reported cut-off values rather than the within-study HR estimates. However, because the studies used study-specific cut-offs, differences in patient classification may remain, and cut-off values should therefore be interpreted in the context of the formula used in each study.

Among the three studies using the scaled formulation, reported cut-off values were 0.12, 0.16, and 0.322, respectively. The cut-off values were determined using different approaches, including maximal log-rank statistics, the Youden index, and cohort-based distributional criteria. The two studies from Hirata et al. reported cut-off values of 1.28 (for PFS in pT3 cohort), and 2.163 (for PFS) and 3.399 (for OS) in the pT1/T2 cohort using the non-scaled formulation. The timing of CALLY assessment also differed across studies. The two localized RCC cohorts measured CALLY postoperatively, whereas the three metastatic RCC cohorts measured CALLY before initiation of systemic therapy. Thus, CALLY was evaluated in different clinical contexts across the included studies.

All five studies reported HRs for both PFS and OS. Multivariable-adjusted estimates were available for all outcomes except the PFS estimate from Hirata et al. (12), which was derived from a univariable analysis. The multivariable models generally adjusted for relevant clinicopathological characteristics and established prognostic factors, although the specific covariates varied across studies. Detailed study characteristics are presented in **Table 1**.

**Table 1:** Characteristics of studies included in the meta-analysis.

Study	Study design	Sample size	Cut-off value (method)	Histology	Stage	Timing of CALLY assessment	Treatment	Adjustment variables	HR for PFS (95% CI)	HR for OS (95% CI)
Hirata et al., 2024 (10)	Retrospective cohort study	80	1.28 (median)	ccRCC	pT3 (any N/M, including T3a/b/c)	Post−operative, 1−week after nephrectomy	Partial or radical nephrectomy	Sex, age, BMI, T stage (T3a vs T3b/c), N stage, M stage, Fuhrman grade (4 vs 1–3), sarcomatoid feature, tumor necrosis	7.71 (2.64–22.50)	3.03 (0.79–11.63)
Akgüner et al., 2025 (11)	Retrospective multicenter cohort study	95	0.12 (maximal log-rank statistics)	Mixed	Metastatic	Pre−treatment baseline, within 1 week prior to initial evaluation	Systemic therapy	KPS (<80%), polymetastatic disease, IMDC risk group, nephrectomy, histology, Fuhrman grade, TNM stage, bone/liver/lung/brain/lymph node metastasis	2.50 (1.45–4.35)	2.44 (1.32–4.55)
Hirata et al., 2025 (12)	Retrospective single-center cohort study	253	PFS: 2.163 OS: 3.399 (Youden index/ROC)	ccRCC	pT1/T2 (N0M0)	Post−operative, at least 1−month after nephrectomy	Partial or radical nephrectomy	T stage (T2 vs T1), Fuhrman grade (3–4 vs 1–2)	3.46 (1.45–8.24) [univariable]	1.77 (0.22–14.44)
Balci et al., 2026 (13)	Retrospective single-center cohort study	68	0.16 (median)	Mixed	Metastatic	Pre−treatment baseline before first−line VEGF−TKI initiation	First-line VEGF-TKI	ECOG PS, IMDC risk, histology, LVI, nephrectomy, lymph node metastasis	1.91 (0.98–3.33)	2.89 (1.36–6.15)
Gurbuz et al., 2026 (14)	Retrospective single-center cohort study	91	0.322 (Youden index/ROC)	Mixed	Metastatic	Pre−treatment baseline prior to nivolumab administration	Nivolumab (2nd line or later in most)	Age group, BMI group, primary tumor surgery, MSKCC risk score, IMDC risk score	2.63 (1.44–4.80)	1.88 (1.05–3.39)

CALLY, C−reactive protein−albumin−lymphocyte index; ccRCC, clear cell renal cell carcinoma; PFS, progression−free survival; OS, overall survival; HR, hazard ratio; CI, confidence interval; IMDC, International Metastatic RCC Database Consortium; ECOG PS, Eastern Cooperative Oncology Group performance status; LVI, lymphovascular invasion; MSKCC, Memorial Sloan Kettering Cancer Center; VEGF−TKI, vascular endothelial growth factor–tyrosine kinase inhibitor; NR, not reported. PFS was defined as time from nephrectomy to recurrence or death in localized ccRCC cohorts, and as time from initiation of systemic therapy to progression or death in metastatic cohorts. These definitions are not directly equivalent.

All five studies were assessed as high quality according to the NOS, with scores ranging from 8 to 9 stars. Hirata et al. (12) received one fewer comparability star because of adjustment for a smaller set of confounders, whereas the other four studies achieved full scores across the assessed domains. Detailed quality assessments are provided in **Supplementary Table 2**.

### CALLY level and prognosis in RCC

All five included studies reported HRs for PFS. The pooled HR for low *versus* high CALLY index was 2.89 (95% CI: 1.82–4.58; *I²* = 24.7%; **Figure 2A**), indicating that patients with a low CALLY index had a significantly higher risk of disease progression than those with a high CALLY index. The between-study variance was τ² = 0.1441, and the 95% prediction interval was 0.84–9.98, which included the null value of 1.0.

**Figure 2:**
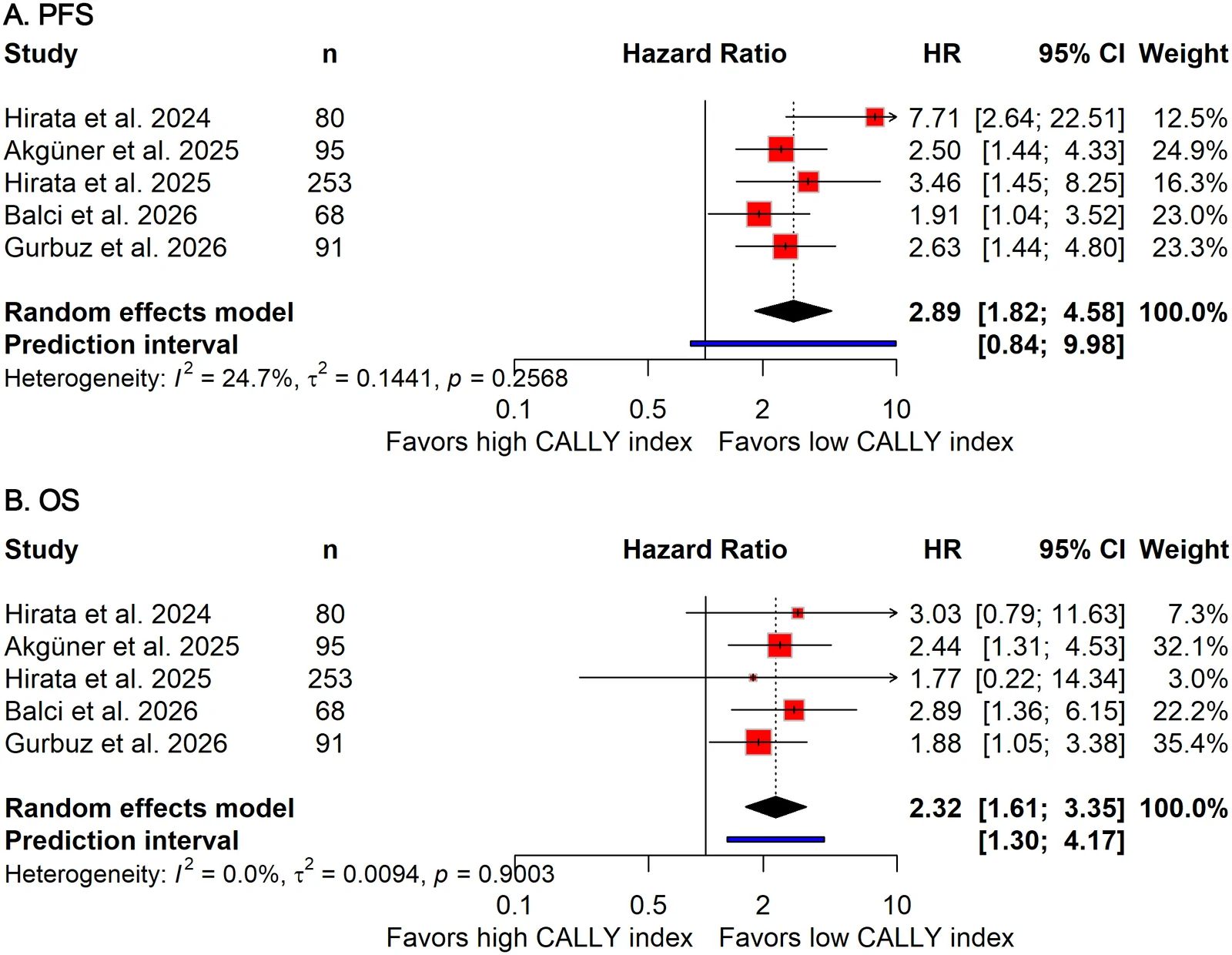
Forest plots showing the association between the CALLY index and progression-free survival **(A)** and overall survival **(B)**.

All five studies also contributed data for OS. The pooled HR was 2.32 (95% CI: 1.61–3.35; *I²* = 0.0%; **Figure 2B**), indicating that a low CALLY index was significantly associated with poorer OS. For OS, τ² = 0.0094, and the 95% prediction interval was 1.30–4.17, which did not include the null value.

Although statistical heterogeneity was low for both outcomes, the prediction interval for PFS included the null value, and these estimates should therefore be interpreted in the context of the small number of studies and the clinical differences in disease stage, treatment setting, timing of CALLY assessment, histological composition, and cut-off determination.

### Subgroup analyses of localized/ccRCC *versus* metastatic/mixed-histology RCC populations

To explore whether the prognostic association of the CALLY index differed across clinically distinct populations, subgroup analyses were conducted according to disease stage and histological composition. Because localized cohorts exclusively comprised ccRCC whereas metastatic cohorts included mixed histologies, disease stage and histological composition were completely concordant across the available studies. Therefore, these analyses should be interpreted as comparisons between localized/ccRCC and metastatic/mixed-histology populations, rather than as independent assessments of disease stage or histology.

For PFS, the pooled HR was 4.89 (95% CI: 2.11–11.32) in the localized/ccRCC subgroup and 2.34 (95% CI: 1.65–3.31) in the metastatic/mixed-histology subgroup. Both subgroups demonstrated statistically significant associations between a low CALLY index and worse PFS. However, the difference between subgroup estimates was not statistically significant (*P* for subgroup difference = 0.112). Given the small number of contributing studies, this exploratory finding should be interpreted with caution and does not establish differential prognostic utility by stage or histology.

For OS, the pooled HR was 2.59 (95% CI: 0.82–8.11) in the localized/ccRCC subgroup and 2.30 (95% CI: 1.56–3.39) in the metastatic/mixed-histology subgroup. The point estimates were directionally consistent, although the localized/ccRCC estimate was imprecise and its CI included the null value, reflecting the limited precision owing to the small number of contributing studies. There was no evidence of a statistically significant subgroup difference (*P* for subgroup difference = 0.847).

Importantly, because disease stage, histological composition, and treatment setting were closely aligned across the available studies, these subgroup analyses do not establish effect modification by any single characteristic. The results are therefore considered exploratory. The subgroup analyses are presented in **Figure 3**.

**Figure 3:**
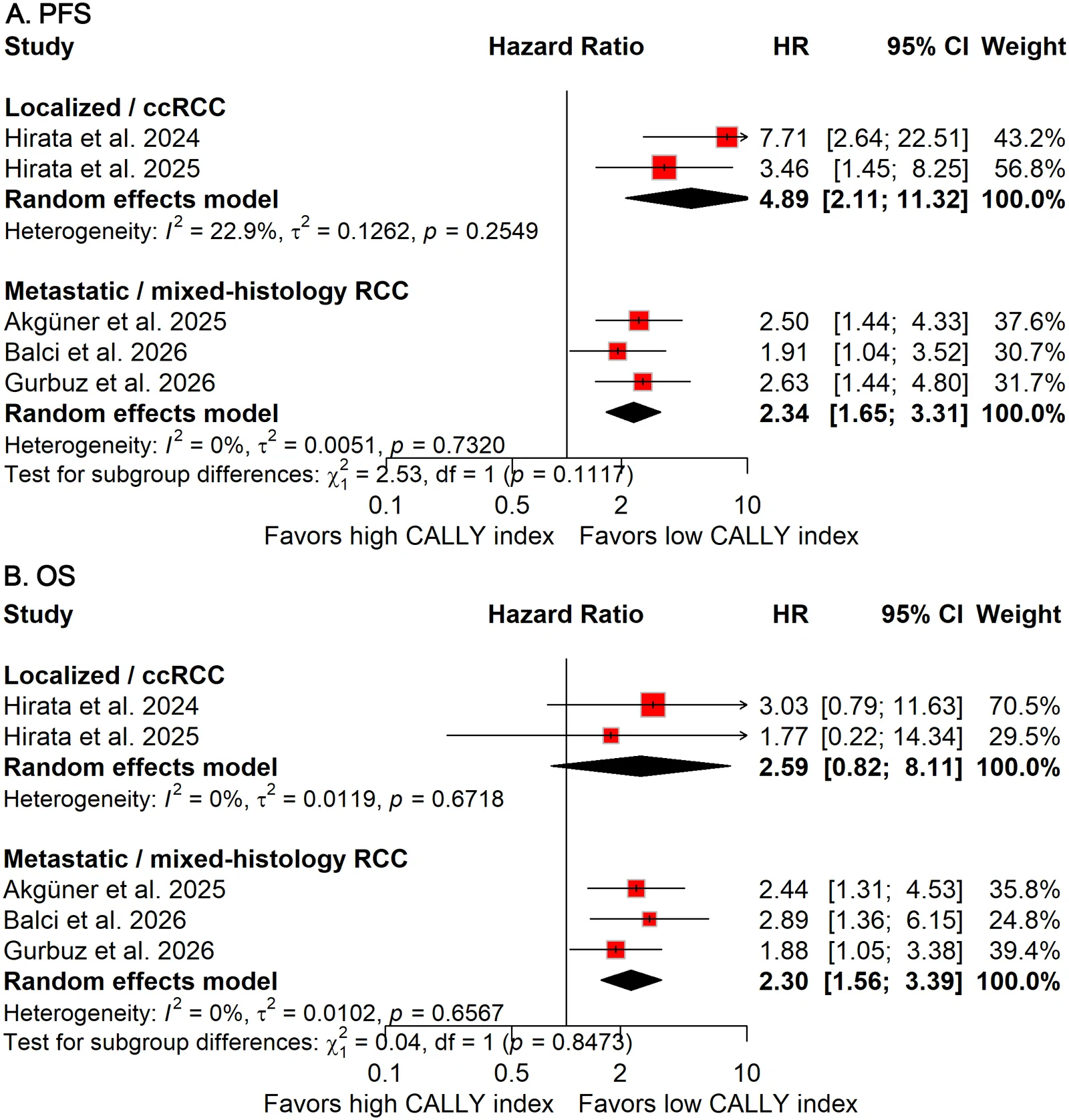
Subgroup analyses of the association between the CALLY index and progression-free survival **(A)** and overall survival **(B)**, stratified according to disease stage and histology-defined populations.

### Sensitivity analysis

Sequential exclusion of individual studies did not materially alter the direction or statistical significance of the pooled associations for either PFS or OS (**Figure 4**). The pooled estimates remained above the null value throughout the analyses, supporting the stability of the overall findings.

**Figure 4:**
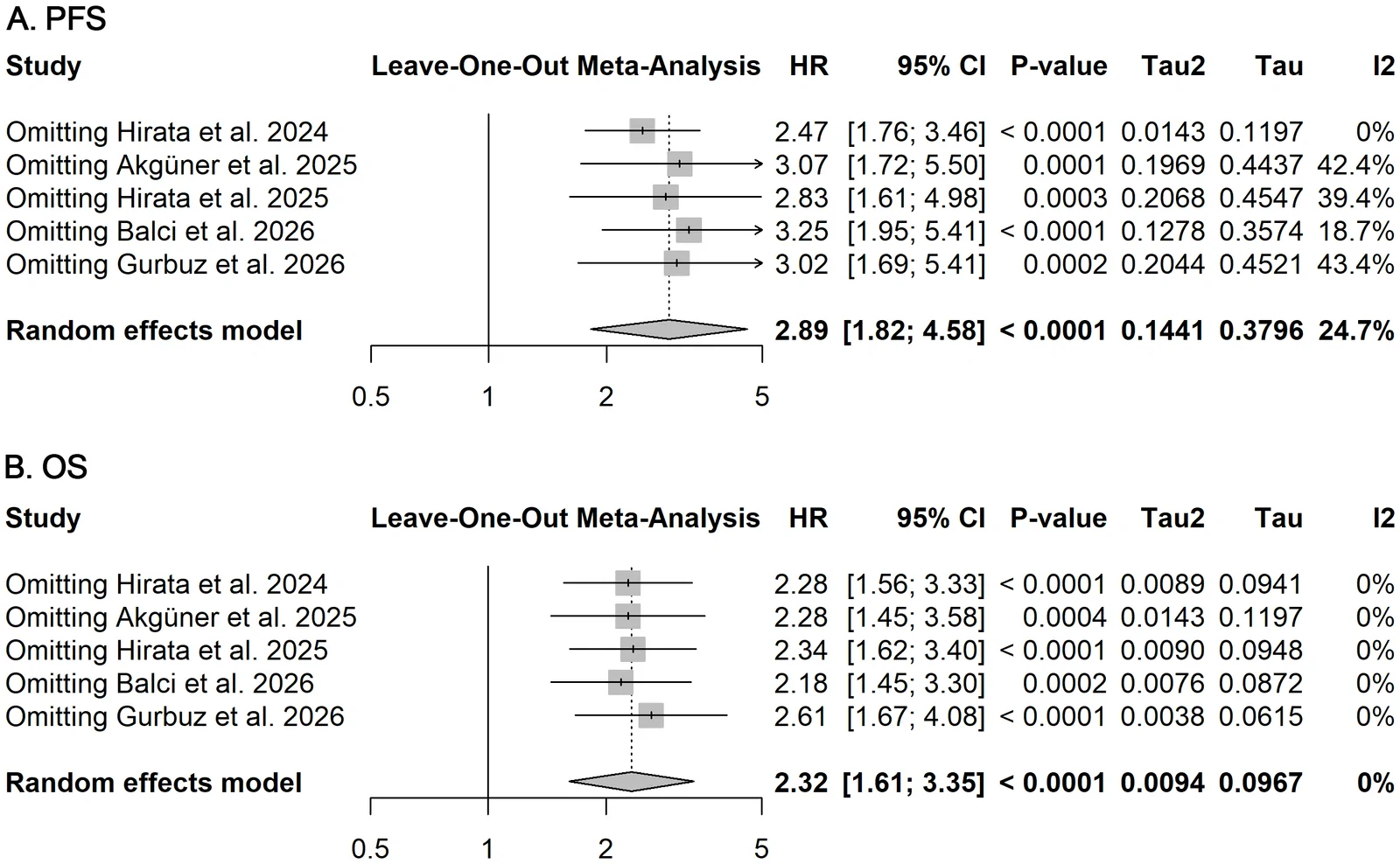
Leave-one-out sensitivity analyses for progression-free survival **(A)** and overall survival **(B)**.

### Publication bias and small-study effects

For PFS, the funnel plot showed a relatively symmetrical distribution of studies around the pooled effect estimate (**Figure 5A**). Exploratory Egger’s regression test yielded a *P* value of 0.056; however, this test was based on only five studies and therefore had limited statistical power. The result should not be interpreted as definitive evidence for or against small-study effects. For OS, the funnel plot showed a broadly symmetrical distribution, and exploratory Egger’s regression test yielded a *P* value of 0.747, providing no statistical evidence of funnel plot asymmetry (**Figure 5B**). However, given the small number of included studies, the absence of statistical evidence should not be interpreted as evidence that publication bias is absent.

**Figure 5:**
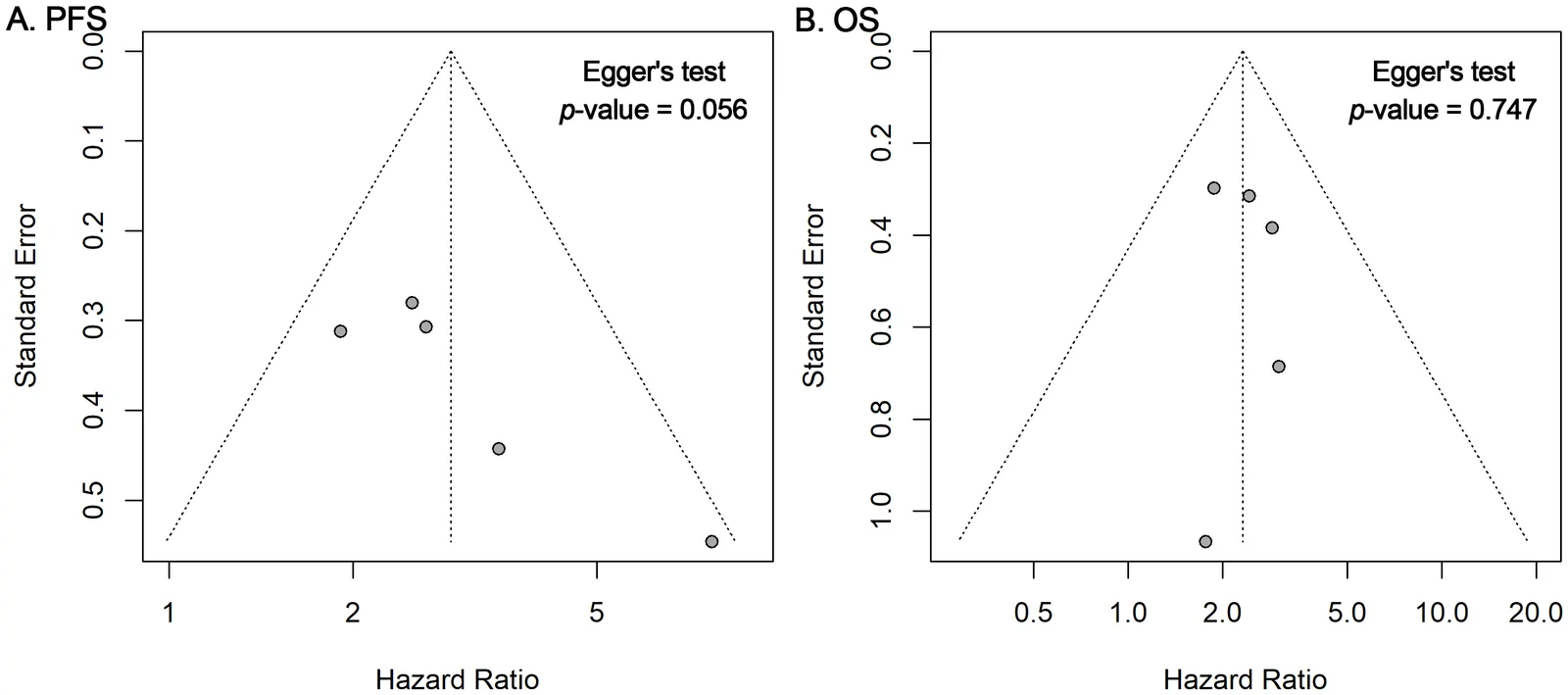
Funnel plots and exploratory Egger’s regression tests for small-study effects in progression-free survival **(A)** and overall survival **(B)**.

## Discussion

To our knowledge, this is the first systematic review and meta-analysis to specifically synthesize the prognostic evidence for the CALLY index in patients with RCC. By pooling five retrospective cohort studies comprising 587 patients, we found that a low CALLY index was significantly associated with worse PFS and OS. The direction of the association was consistent across the included studies and remained stable in leave-one-out sensitivity analyses. These findings suggest that CALLY may capture clinically relevant aspects of host systemic status in RCC and may have potential as an adjunctive prognostic biomarker. Nevertheless, the evidence should be interpreted cautiously because all available studies were retrospective and differed in disease stage, treatment setting, timing of CALLY assessment, numerical scaling, and cut-off determination.

A recent umbrella meta-analysis by Zhao et al. (15), encompassing 56 reports across diverse cancer types, reported a pooled HR of 0.55 for OS in favor of a high CALLY index, with a subgroup analysis of urological cancers yielding a pooled HR of 0.41. However, only three RCC-specific comparison groups were included in that analysis, limiting the ability to characterize RCC-specific clinical settings. Our study complements this broader evidence by focusing specifically on RCC and incorporating more recent RCC cohorts. Importantly, the present analysis highlights methodological and clinical characteristics that may be particularly relevant when interpreting the RCC-specific evidence. Although the magnitude of the observed associations appears clinically meaningful, HRs derived from separate meta-analyses should not be directly compared because of differences in patient populations, endpoint definitions, CALLY cut-off definitions, timing of measurement, and covariate adjustment. Direct comparisons within the same patient populations will be required to determine whether CALLY provides incremental prognostic information beyond established inflammatory, nutritional, and clinical risk factors.

The biological plausibility of the CALLY index in RCC derives from its integration of three complementary dimensions of host-tumor interaction: systemic inflammation, nutritional status, and immune competence. CRP reflects systemic inflammatory activity, which may promote tumor progression through cytokine signaling, angiogenesis, and immune dysregulation (19). In RCC, these processes occur within a tumor microenvironment characterized by complex angiogenic and immune interactions, including dysregulation of the von Hippel–Lindau/hypoxia-inducible factor pathway (13, 14). Serum albumin is influenced by both nutritional reserve and inflammation-associated metabolic changes, whereas lymphopenia may reflect impaired antitumor immune surveillance (20, 21). The combination of these parameters may therefore capture multiple aspects of host condition that are incompletely represented by single-component biomarkers.

However, the biological interpretation of CALLY should remain cautious. Each component is nonspecific and can be influenced by factors unrelated to tumor biology, including infection, inflammatory comorbidities, nutritional status, organ function, and treatment-related changes. Accordingly, CALLY should currently be viewed as a prognostic composite marker rather than a direct mechanistic measure of tumor immunity or inflammation.

The pooled HRs for PFS and OS indicate that patients classified as having a low CALLY index had approximately threefold and twofold higher risks of progression and death, respectively, than patients with a high CALLY index. Most underlying estimates were derived from multivariable models, suggesting that the observed associations were not entirely explained by the clinicopathological variables included in those models. However, the covariates differed among studies, and one PFS estimate was based on a univariable analysis. Residual confounding therefore cannot be excluded. Moreover, a prognostic association does not establish that CALLY itself has a causal role in disease progression or treatment response. Rather, CALLY may function as an accessible marker of the broader systemic condition of the patient.

An important source of clinical heterogeneity is the timing of CALLY assessment. The two localized RCC studies measured CALLY after nephrectomy, whereas the three metastatic studies measured CALLY before systemic treatment. These measurements are therefore not directly equivalent. In particular, an early postoperative CALLY value may be influenced by surgical trauma, acute inflammatory responses, fluid shifts, and transient nutritional changes. A measurement obtained later after surgery may better reflect recovery and baseline host status. By contrast, pretreatment CALLY in metastatic disease may reflect systemic inflammatory burden, nutritional reserve, immune competence, and potentially tumor burden immediately before systemic therapy. Thus, CALLY may represent different biological states depending on the clinical context. This distinction is important because the current pooled estimate combines postoperative and pretreatment measurements. Although the direction of the association was consistent, the available evidence is insufficient to determine whether CALLY has the same prognostic meaning across these different time points.

The numerical CALLY cut-offs also differed across studies. Two studies used a formulation without the 10^4^ scaling factor, whereas three studies used the formulation incorporating 10^4^ in the denominator. When laboratory units are otherwise equivalent, these formulations are mathematically related by a fixed scaling factor. Therefore, numerical cut-off values from the two conventions should not be interpreted as directly different biological thresholds. Importantly, even among studies using the same scaling convention, cut-off values differed. The standardized formulation yielded reported cut-offs of 0.12, 0.322, and 0.16 across the three relevant studies. These values were derived using different approaches, including maximal log-rank statistics, the Youden index, and cohort-based criteria. Therefore, cut-off heterogeneity cannot be attributed solely to formula scaling. This lack of standardization currently represents an important barrier to clinical implementation. A numerical cut-off derived from a single cohort may perform differently in another population because of differences in disease stage, treatment, laboratory characteristics, and outcome distributions. Future studies should therefore prespecify and externally validate RCC-specific CALLY thresholds rather than repeatedly deriving study-specific optimal cut-offs.

Despite the above differences, statistical heterogeneity was relatively low in the primary analyses, with *I²* values of 24.7% for PFS and 0.0% for OS. This apparent consistency is noteworthy but should not be interpreted as evidence that the underlying populations were clinically equivalent. Heterogeneity statistics are imprecise when only a small number of studies are available and may fail to identify clinically meaningful differences between study populations. In the present analysis, localized and metastatic cohorts differed in disease burden, treatment modality, timing of CALLY measurement, and histological composition. The low *I²* values therefore indicate that the observed study-level effect estimates were relatively consistent, but they do not eliminate concerns regarding clinical and methodological heterogeneity. Consistent with this concern, the 95% prediction interval for PFS extended below 1.0 (0.84–9.98), indicating that the prognostic association may not be uniformly observed in future clinical settings. In contrast, the prediction interval for OS remained above 1.0 (1.30–4.17), suggesting that the association with overall survival is more consistent across populations. This divergence is likely attributable to the higher between-study variance for PFS (τ² = 0.1441) compared with OS (τ² = 0.0094), which in turn reflects the clinical and methodological heterogeneity of the PFS endpoint—particularly the different event definitions (postoperative recurrence vs. progression during systemic therapy) and the wider range of cut-off values used across studies. The wider prediction interval in the overall PFS analysis may be largely attributable to the localized studies, which were fewer in number and exhibited greater between-study variability (*I²* = 22.1%).

The subgroup analyses provide additional context but should be interpreted cautiously. For PFS, the pooled association appeared numerically stronger in the localized/ccRCC subgroup than in the metastatic/mixed-histology subgroup, with HRs of 4.89 and 2.34, respectively. However, the difference between subgroups was not statistically significant (*P* for interaction = 0.112). More importantly, disease stage, histological composition, and treatment setting were completely or closely aligned across the available studies. Consequently, the observed difference cannot be attributed specifically to disease stage, histology, or treatment modality. The larger point estimate in localized disease could reflect postoperative measurement timing, differences in recurrence biology, exclusive inclusion of ccRCC, differences in cut-off determination, sample size, or other study-level characteristics. The finding should therefore be regarded as exploratory rather than evidence that CALLY has a stronger prognostic effect in localized RCC. For OS, the pooled estimates were directionally similar in the localized/ccRCC and metastatic/mixed-histology subgroups, at 2.59 and 2.30, respectively, with no evidence of a subgroup difference (*P* for interaction = 0.847). The wide confidence interval for the localized/ccRCC estimate reflects the small number of contributing studies and limited precision. Although the two localized studies individually yielded non-significant OS associations, their pooled estimate remained directionally consistent with the metastatic studies. This pattern is compatible with a potentially shared prognostic association across clinical settings, but the available evidence is insufficient to establish equivalence.

From a clinical perspective, the CALLY index has several practical advantages. It is calculated using CRP, serum albumin, and absolute lymphocyte count, all of which are routinely available during the clinical evaluation of patients with RCC. It requires no specialized equipment or additional laboratory infrastructure and may therefore be feasible across diverse healthcare settings. The CALLY index could potentially complement established prognostic frameworks, particularly in metastatic RCC, where the International Metastatic Renal Cell Carcinoma Database Consortium (IMDC) and Memorial Sloan Kettering Cancer Center (MSKCC) criteria are widely used. Several included studies reported associations between CALLY and survival after adjustment for established prognostic variables or risk scores, suggesting that CALLY may capture aspects of host systemic condition not completely represented by conventional clinical factors. However, the current evidence does not support using CALLY alone to select systemic therapy, change surveillance schedules, or make treatment decisions. Demonstrating clinical utility requires more than statistical association. Future studies should determine whether adding CALLY to established prognostic models improves discrimination, calibration, risk reclassification, and clinical decision-making.

Several limitations should be considered. First, all included studies were retrospective, inherently introducing potential selection bias, information bias, and residual confounding (22–24). All estimates except the PFS estimate from Hirata et al. (12) were derived from multivariable models; however, the covariates included in these models differed across studies. Second, CALLY was measured at different clinical time points, including early postoperative, later postoperative, and pretreatment settings. This variation may affect the biological interpretation of the index and limits direct comparability across studies. In addition, the PFS endpoint pooled different event definitions—postoperative recurrence in localized disease versus progression during systemic therapy in metastatic disease—which may affect the interpretability of the pooled estimate. Third, two numerical scaling conventions were used, and cut-off determination methods varied substantially. Although the underlying scaled and non-scaled formulations are mathematically related when laboratory units are equivalent, the absence of a validated common threshold remains an important limitation for clinical translation. Fourth, subgroup analyses were based on only two localized studies and three metastatic studies. Disease stage, histological composition, and treatment setting were closely aligned, preventing reliable disentanglement of their individual effects. More granular analyses according to treatment modality, histological subtype, or metastatic site were therefore not statistically reliable. Fifth, only five studies were available, precluding robust meta-regression and limiting the reliability of formal assessments of publication bias. In particular, the borderline *P* value for Egger’s test for PFS should not be interpreted as definitive evidence of publication bias or small-study effects. The 95% prediction interval for PFS included the null value (0.84–9.98), indicating that the pooled estimate should be interpreted with caution when extrapolated to future populations. Sixth, although all included studies achieved high NOS scores, the NOS primarily evaluates the methodological quality and reporting of observational studies and does not capture unmeasured confounding or guarantee causal inference. Finally, all included studies evaluated CALLY at a single time point. The prognostic significance of longitudinal CALLY changes and whether dynamic changes provide additional information beyond baseline CALLY therefore remain unknown.

Future research should prioritize large, prospective, multicenter cohorts using standardized protocols for blood sampling, laboratory measurement, CALLY calculation, and cut-off definition. The timing of CALLY assessment should be prespecified because postoperative and pretreatment measurements may represent different biological states. External validation of prespecified CALLY thresholds is particularly important. Rather than repeatedly selecting cohort-specific optimal cut-offs, future studies should evaluate whether previously defined thresholds maintain prognostic performance in independent populations. The prognostic value of serial CALLY measurements and changes over time also warrants investigation, particularly among patients receiving systemic therapy. Furthermore, the incremental value of CALLY beyond established prognostic models, including IMDC, MSKCC, and the Leibovich score, should be evaluated using measures of discrimination, calibration, reclassification, and clinical utility. Future studies should also determine whether CALLY provides prognostic or predictive information within specific treatment settings, particularly immune checkpoint inhibitor-based regimens, and in non-ccRCC subtypes, which remain underrepresented in the current literature. Mechanistic studies may further clarify how systemic inflammation, nutritional status, and immune dysfunction interact in RCC and whether CALLY reflects biologically meaningful host-tumor interactions.

## Conclusion

This systematic review and meta-analysis indicates that a low CALLY index is associated with poorer PFS and OS across the heterogeneous RCC populations represented in the available retrospective studies. These pooled estimates should be interpreted as overall prognostic associations rather than as evidence of stage-specific or treatment-specific effects. The direction of the association was consistent across localized/ccRCC and metastatic/mixed-histology populations, although the apparent difference in the magnitude of the PFS association was not statistically significant and cannot be attributed specifically to disease stage or histology because these characteristics were closely aligned across the available studies. The CALLY index is a simple and readily available composite biomarker that may provide complementary prognostic information in RCC. However, the current evidence is limited by retrospective study designs, heterogeneous clinical settings, differences in CALLY measurement timing and numerical scaling, variable cut-off determination, and the absence of externally validated thresholds. Prospective multicenter studies with standardized measurement protocols and validated cut-off values are warranted to determine the incremental prognostic value and clinical utility of the CALLY index in RCC.

## Data Availability

The datasets presented in this study can be found in online repositories. The names of the repository/repositories and accession number(s) can be found in the article/**Supplementary Material**.
