# Supplementary material for: C-reactive protein-albumin-lymphocyte index and survival outcomes in renal cell carcinoma: a systematic review and meta-analysis
Source: Front Oncol. 2026 Oct 27;16:1994071. doi: 10.3389/fonc.2026.1994071 (PMC13747836; doi:10.3389/fonc.2026.1994071)
Supplement: Supplementary file 1 [file Supplementaryfile1.docx]

**Supplementary Material**

**C: a systematic review and meta-analysis**

**Supplementary Table 1.** Details of search strategy.

**Supplementary Table 2.** Quality assessment of included studies through the Newcastle-Ottawa Scale.

**Supplementary Table 1.** Details of search strategy.

| **Database** | **Search strategy** |
| --- | --- |
| ***PubMed*** | ("Carcinoma, Renal Cell"[Mesh] OR "renal cell carcinoma" OR "renal cancer" OR "kidney cancer") AND (CALLY OR "C-reactive protein albumin lymphocyte" OR "C-reactive protein albumin lymphocyte index" OR "C-reactive protein-albumin-lymphocyte index")  **Search items: 8**  **(initial: August 20, 2026, updated October 10, 2026)** |
| ***Embase*** | ('renal cell carcinoma'/exp OR 'renal cell carcinoma' OR 'renal cancer' OR 'kidney cancer') AND (cally OR 'c reactive protein albumin lymphocyte' OR 'c-reactive protein albumin lymphocyte')  **Search items: 24 (August 20, 2026)**  **(updated October 10, 2026: 25 search items)** |

**Supplementary Table 2.** Quality assessment of included studies through the Newcastle-Ottawa Scale.

| **Study** | **Selection (0–4)** | **Comparability (0–2)** | **Outcome (0–3)** | **Total (0–9)** | **Quality** |
| --- | --- | --- | --- | --- | --- |
| Hirata et al. 2024 | ⭐⭐⭐⭐ (4) | ⭐⭐ (2) | ⭐⭐⭐ (3) | 9 | High |
| Akgüner et al. 2025 | ⭐⭐⭐⭐ (4) | ⭐⭐ (2) | ⭐⭐⭐ (3) | 9 | High |
| Hirata et al. 2025 | ⭐⭐⭐⭐ (4) | ⭐ (1) | ⭐⭐⭐ (3) | 8 | High |
| Balci et al. 2026 | ⭐⭐⭐⭐ (4) | ⭐⭐ (2) | ⭐⭐⭐ (3) | 9 | High |
| Gurbuz et al. 2026 | ⭐⭐⭐⭐ (4) | ⭐⭐ (2) | ⭐⭐⭐ (3) | 9 | High |
